# Metabolic and nutritional impact of gastrectomy and perioperative treatment in gastric cancer: a prospective cohort study

**DOI:** 10.1186/s43046-026-00367-6

**Published:** 2026-06-22

**Authors:** Tomasz Muszyński, Tomasz Jędrychowski, Adrianna Witalewska, Aldona Gawlewicz-Czepiel, Karina Polak, Michał Spieszny, Piotr Richter, Antoni Szczepanik, Kołodziejczyk Piotr

**Affiliations:** 1 Medical College, Doctoral School of Medical and Health Sciences, Jagiellonian University, St. Anne Street 11, Krakow, 31-008 Poland; 2 Medical College, Ist Chair of General Surgery, Department of General, Oncological, Gastrointestinal Surgery and Transplantology, University Hospital in Krakow, Jagiellonian University, Jakubowskiego 2, Krakow, 30-688 Poland; 3 Medical College, Jagiellonian University, Krakow, Poland; 4 Chair and Department of Dermatology, Medical University of Silesia, Francuska Street 20-24, Katowice, 40-027 Poland; 5 Institute of Sports Sciences, University of Physical Culture in Krakow, Krakow, Poland; 6 Medical College, 3rd Chair of General Surgery, Jagiellonian University, Pradnicka Street 35, Krakow, 31- 202 Poland

**Keywords:** Stomach neoplasms, Gastrectomy, Nutritional status

## Abstract

**Background:**

Gastrectomy due to gastric cancer leads to metabolic alterations in body composition, significantly affecting nutritional status. This prospective study aimed to investigate the metabolic effects of gastrectomy and perioperative treatment on the nutritional status of gastric cancer patients.

**Methods:**

A total of 37 patients diagnosed with gastric cancer, treated with perioperative chemotherapy, without evidence of dissemination or metastasis, were included in the study. Demographic data, Body Mass Index, full blood count parameters, lipid profiles, vitamin B12, vitamin D, iron levels, albumin, prealbumin, total serum protein concentration, bioimpedance parameters, physical activity levels, quality of life, Prognostic Nutritional Index, neutrophil-to-lymphocyte ratio, and systemic immune-inflammation index were assessed preoperatively and at two follow-ups (6 and 12 months post-surgery) for each patient.

**Results:**

Significant changes were observed across the analyzed parameters, demonstrating the metabolic impact of gastrectomy and perioperative systemic treatment on the nutritional status of gastric cancer patients.

**Conclusions:**

The study provides a comprehensive evaluation of the long-term nutritional status of gastric cancer patients post-gastrectomy, offering valuable insights into postoperative metabolic and dietary adaptations. Obtained results underscore the dynamic physiological adaptations following gastric resection, emphasizing the importance of nutritional and metabolic monitoring for long-term recovery. Targeted rehabilitation strategies and micronutrient supplementation are crucial in reducing postoperative complications and improving patient outcomes.

**Supplementary Information:**

The online version contains supplementary material available at 10.1186/s43046-026-00367-6.

## Background

### Introduction

Gastric cancer causes ~ 800,000 deaths annually, ranking fifth in global prevalence and third in cancer mortality [1]. Malnutrition impacts ~ 60% of patients, worsening outcomes and quality of life [2]. Perioperative chemotherapy is now standard in Europe [3], but may impair nutritional status, especially after surgery. Despite this, few studies have explored perioperative nutrition and its impact on recovery [4]. This prospective study investigates metabolic and nutritional changes to enhance patient care.

## Methods

A prospective cohort study (2021–2024) was conducted at the Jagiellonian University Medical College and University Hospital in Kraków, enrolling 37 patients with locally advanced gastric cancer treated per Polish guidelines [3]. Ethical approval was obtained (Jagiellonian University Ethics Committee, No. 1072.6120.60.2021), and all participants gave informed consent. Inclusion and exclusion criteria are listed in Table 1.

**Table 1:** Study inclusion and exclusion criteria

Inclusion criteria	Exclusion criteria
1. A confirmed clinical and histopathological diagnosis of gastric cancer	1. Lack of informed consent
2. Absence of clinical evidence of tumor dissemination or metastasis	2. Pregnancy or lactation
3. Age ≥ 18 years	3. WHO performance status > 2
4. Receipt of adjuvant chemotherapy during the perioperative period	4. Presence of chronic comorbid conditions that, in the authors' opinion, could significantly impact nutritional status (e.g., inflammatory bowel diseases)

**Fig. 1:**
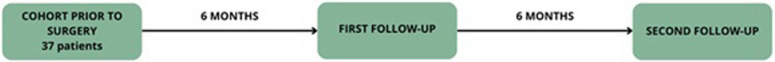
Study design timeline

Most patients received chemotherapy—preoperatively (7), perioperatively (21), or postoperatively (5)—while 2 had early gastric cancer and 2 declined treatment; notably, the predominance of perioperative therapy represents a key study novelty reflecting current practice. Nineteen patients received FLOT (5-fluorouracil, leucovorin, oxaliplatin, and docetaxel) chemotherapy; postoperatively, 12 continued FLOT, while 7 were treated locally with unspecified regimens. Patients were assessed prior to gastrectomy and during routine oncological follow-ups at defined intervals: the day before surgery, six months postoperatively, and every six months thereafter; the study design timeline is presented in Fig. 1. Postoperative chemotherapy usually begins ~ 1 month after surgery, lasting up to 3 months [5]; thus, follow-up was scheduled at 6 months. After total gastrectomy, vitamin B12 was routinely supplemented (dose varied), iron as required, while vitamin D was not routinely prescribed.

Evaluated parameters included blood counts, lipid profiles, vitamin B12, vitamin D, iron, albumin, prealbumin, total serum protein, bioimpedance metrics, physical activity, and quality of life. Nutritional status was assessed using selected cut-off values: serum iron < 70 μg/L [6], vitamin B12 < 197.0 pg/mL [7], vitamin D < 30 nmol/L [8] and others presented in Table 2. Bioimpedance was assessed using the TANITA SC-240 analyzer, measuring BMI, body weight, and body composition parameters, including fat mass, fat-free mass, total body water, muscle and bone mass, metabolic age, and visceral fat (≥ 13 indicating high accumulation) [13]. Sarcopenia was assessed using SMI (kg/m^2^), with moderate defined as 8.51–10.75 in men and 5.76–6.75 in women, and severe as ≤ 8.50 and ≤ 5.75, respectively [14]. Physical activity was evaluated using the IPAQ-SF and expressed in METs (Metabolic Equivalent of Task)/week [15]. Quality of life was measured via the EORTC QLQ-C30 version 3.0 questionnaire. [16, 17]. Statistical analysis was conducted using R software (v.4.3.3), with significance threshold of *p* < 0.05 applied; details are provided in the Supplementary Material 1.

**Table 2:** Selected possible malnutrition indicators investigated in the study with their cut-off values

**Total Lymphocyte Count (TLC)**	**Prealbumin Level**	**Albumin Level**	**Total Protein Level**	**Total Cholesterol Level**
No malnutrition: > 1,500 [10⁹/L] Mild malnutrition: 1,200–1,499 [10⁹/L] Moderate malnutrition: 800–1,199 [10⁹/L] Severe malnutrition: < 800 [10⁹/L][9]	No malnutrition: > 18 mg/dL Mild malnutrition: 10–17 mg/dL [10]	Normal: > 3.5 g/L [9]	Normal: > 60 g/L [11]	Normal: > 2.5863 mmol/L Malnutrition: < 2.5863 mmol/L [12]

## Results

### Study group

Out of 37 patients, 26 underwent total and 11 subtotal gastrectomy; the groups showed no significant differences in baseline characteristics (age, sex, BMI, tumor stage, or recurrence).

Median age was 66 years (IQR: 55–72), and 67.57% were male. Tumor staging revealed 48.65% in stage III, 32.43% in stage II, and 18.92% in stage I. Detailed baseline characteristics by gastrectomy type are shown in Table 3.

**Table 3:** Characteristics of demographic and basic health metrics for overall sample and with stratified by the gastrectomy type

Characteristic	N	Overall sample^a^	Resection type		p^c^
			total, n_1_ = 26^a^	subtotal, n_2_ = 11^a^	
Age, years	37	66.00 (55.00, 72.00)^b^	67.00 (60.00, 72.00)^b^	55.00 (48.00, 68.00)^b^	0.096^d^
gender	37				0.279
female		12 (32.43%)	10 (38.46%)	2 (18.18%)	
male		25 (67.57%)	16 (61.54%)	9 (81.82%)	
BMI, kg/m^2^	37	25.83 (23.90, 28.76)^b^	25.59 (23.91, 29.60)^b^	26.61 (23.57, 28.06)^b^	0.635^d^
Relapse	37	8 (21.62%)	4 (15.38%)	4 (36.36%)	0.203
Stage^e^:	37				0.916
IA		2 (5.41%)	2 (7.69%)	0 (0%)	
IB		5 (13.51%)	4 (15.38%)	1 (9.09%)	
IIA		4 (10.81%)	2 (7.69%)	2 (18.18%)	
IIB		8 (21.62%)	5 (19.23%)	3 (27.27%)	
IIIA		8 (21.62%)	6 (23.08%)	2 (18.18%)	
IIIB		10 (27.03%)	7 (26.92%)	3 (27.27%)	

*N* sample size, *n* group size, *Mdn* median, *Q1* the first quartile (25%), *Q3* the third quartile (75%), *p* the *p*-value of statistical test

^a^n (%)

^b^Median (IQR)

^c^Fisher’s exact test

^d^Wilcoxon rank sum test

^e^The 8th edition of the American Joint Committee on Cancer Classification

Table 4 summarizes the assessment results across multiple clinical domains.

**Table 4:** Changes in body composition, quality of life, hematological, nutritional, lipid, micronutrient, and inflammation parameters in patients undergoing stomach resection over 12 months of follow-up

Characteristic	N	Follow up		
		Baseline (pre-operation), n = 37^a^	post-operation (6 months), n = 37^a^	post-operation (12 months), n = 37^a^
Mass, kg	37	76.30 (64.20, 82.89)	64.70 (63.77, 66.09)	74.93 (70.00, 78.62)
BMI, kg/m^2^	37	25.83 (23.90, 28.76)	22.60 (21.38, 24.73)	25.93 (23.06, 27.53)
Fat percentage	37	22.60 (18.30, 26.80)	17.52 (15.35, 19.80)	29.50 (26.06, 32.18)
Fat mass, kg	37	17.20 (13.10, 21.60)	11.43 (9.90, 13.40)	22.60 (19.40, 25.66)
Fat free mass, kg	37	55.80 (50.00, 61.02)	53.02 (52.34, 53.70)	55.08 (52.82, 57.34)
Total body water, kg	37	40.06 (35.45, 43.82)	37.33 (36.73, 37.93)	38.05 (36.87, 39.03)
Predicted muscle mass, kg	37	53.00 (47.50, 57.99)	50.36 (49.70, 51.00)	52.33 (50.16, 54.50)
Bone tissue mass, kg	37	2.80 (2.52, 3.02)	2.66 (2.63, 2.70)	2.76 (2.66, 2.85)
Skeletal muscle mass, kg	37	31.60 (28.30, 34.53)	30.01 (29.62, 30.40)	30.23 (29.02, 31.43)
Visceral fatty tissue	37	10.00 (9.00, 11.00)	7.00 (7.00, 8.00)	11.00 (10.00, 11.00)
Skeletal muscle index	37	10.80 (10.03, 12.08)	10.28 (9.86, 11.09)	10.62 (9.83, 11.42)
Metabolic age, years	37	53.00 (52.00, 54.04)	47.00 (46.00, 51.00)	56.22 (55.75, 56.93)
*Quality of life, sarcopenia and activity*				
QoL	37	62.11 (57.77, 69.48)	73.07 (70.13, 76.64)	69.87 (67.24, 72.07)
Physical activity levels:	37			
active		1 (2.70%)^b^	5 (13.51%)^b^	0 (0%)^b^
minimal		13 (35.14%)^b^	17 (45.95%)^b^	3 (8.11%)^b^
inactive		23 (62.16%)^b^	15 (40.54%)^b^	34 (91.89%)^b^
Total amount of effort in METs per week	37	441.48 (33.00, 767.15)	1,640.83 (1,303.35, 2,162.40)	1,021.86 (824.08, 1,296.77)
Sarcopenia [kg/m2] levels:	37			
no		25 (67.57%)^b^	20 (54.05%)^b^	20 (54.05%)^b^
moderate		12 (32.43%)^b^	16 (43.24%)^b^	15 (40.54%)^b^
severe		0 (0%)^b^	1 (2.70%)^b^	2 (5.41%)^b^
*Hematological parameters*				
WBC	37	7.10 (5.96, 8.38)	6.27 (5.22, 8.09)	7.64 (6.24, 8.41)
LYMPH	37	1.58 (1.40, 2.10)	1.69 (1.45, 2.26)	1.89 (1.76, 1.99)
Hgb	37	12.10 (9.50, 13.30)	12.20 (12.00, 12.50)	12.76 (12.20, 13.20)
Hgb levels:	37			
no anemia		13 (35.14%)^b^	11 (29.73%)^b^	16 (43.24%)^b^
mild anemia		14 (37.84%)^b^	23 (62.16%)^b^	20 (54.05%)^b^
moderate anemia		10 (27.03%)^b^	3 (8.11%)^b^	1 (2.70%)^b^
Hct	37	36.60 (29.70, 39.10)	36.79 (36.70, 38.20)	38.22 (37.30, 38.90)
PLT	37	275.00 (212.00, 347.00)	278.06 (212.00, 310.23)	319.00 (226.00, 354.83)
*Nutritional markers*				
ALB, g/dL	37	4.05 (3.84, 4.33)	3.97 (3.77, 4.36)	4.31 (4.24, 4.39)
ALB levels:	37			
no malnutrition		34 (91.89%)^b^	34 (91.89%)^b^	37 (100%)^b^
mild malnutrition		3 (8.11%)^b^	3 (8.11%)^b^	0 (0%)^b^
PREALB	37	0.21 (0.19, 0.25)	0.21 (0.18, 0.23)	0.20 (0.19, 0.21)
PREALB levels:	37			
no malnutrition		23 (62.16%)^b^	23 (62.16%)^b^	26 (70.27%)^b^
mild malnutrition		14 (37.84%)^b^	14 (37.84%)^b^	9 (24.32%)^b^
moderate malnutrition		0 (0%)^b^	0 (0%)^b^	2 (5.41%)^b^
Total protein	37	64.10 (61.04, 68.60)	65.50 (63.03, 66.60)	68.64 (67.67, 69.70)
Total protein level:	37			
normal level		13 (35.14%)^b^	17 (45.95%)^b^	32 (86.49%)^b^
below normal		24 (64.86%)^b^	20 (54.05%)^b^	5 (13.51%)^b^
PNI	37	48.60 (45.75, 53.15)	48.40 (45.81, 51.95)	52.42 (51.91, 54.42)
PNI levels:	37			
no malnutrition		15 (40.54%)^b^	16 (43.24%)^b^	32 (86.49%)^b^
mild malnutrition		15 (40.54%)^b^	14 (37.84%)^b^	2 (5.41%)^b^
moderate to severe malnutrition		5 (13.51%)^b^	5 (13.51%)^b^	2 (5.41%)^b^
severe malnutrition		2 (5.41%)^b^	2 (5.41%)^b^	1 (2.70%)^b^
*Lipid profile*				
TG	37	1.45 (1.14, 1.74)	1.27 (0.88, 1.66)	1.04 (0.85, 1.39)
CHOL	37	4.21 (3.90, 4.60)	4.13 (3.47, 5.00)	3.74 (3.00, 4.22)
CHOL levels:	37			
no malnutrition		34 (91.89%)^b^	36 (97.30%)^b^	33 (89.19%)^b^
malnutrition		3 (8.11%)	1 (2.70%)	4 (10.81%)
HDL	37	1.27 (1.07, 1.32)	1.30 (1.20, 1.36)	1.40 (1.34, 1.46)
LDL calculated	37	2.52 (2.10, 2.63)	2.54 (2.20, 3.04)	2.03 (1.59, 2.47)
*Micronutrients*				
Fe	37	9.34 (5.76, 14.25)	7.35 (5.03, 10.20)	12.88 (12.12, 13.50)
Vitamin B12	37	1,136.09 (416.00, 1,576.51)	2,406.36 (736.36, 3,645.60)	400.29 (365.00, 431.89)
Vitamin D	37	21.84 (14.40, 22.50)	24.44 (22.46, 26.22)	33.04 (21.07, 42.01)
*Inflammation and immune markers*				
NLR	37	2.83 (2.06, 3.62)	2.33 (1.41, 3.08)	2.59 (1.90, 2.84)
NLR levels:	37			
high NLR		27 (72.97%)^b^	22 (59.46%)^b^	24 (64.86%)^b^
low NLR		10 (27.03%)^b^	15 (40.54%)^b^	13 (35.14%)^b^
SII, cells/L	37	778.99 (519.34, 1,064.77)	684.20 (498.90, 796.19)	813.79 (515.39, 995.91)
SII levels:	37			
high SII		25 (67.57%)^b^	22 (59.46%)^b^	23 (62.16%)^b^
low SII		12 (32.43%)^b^	15 (40.54%)^b^	14 (37.84%)^b^

*Abbreviations:* *BMI* Body mass index, *QoL* quality of life, *METs* Metabolic Equivalent of Task, *WBC* white blood count, *LYMPH* lymphocytes level, *Hgb* hemoglobin level, *Hct* hematocrit, *PLT* platelet count, *ALB* albumin level, *PREALB* prealbumin level, *PNI* prognostic nutritional index, *TG* triglicerydes, *CHOL* cholesterol, *HDL *high-density lipoprotein, *LDL *low-density lipoprotein, *Fe* iron, *NLR *neutrophil-to-lymphocyte ratio, *SII *The systemic immune-inflammation index

^a^ Mdn (Q1, Q3)

^b^ n (%)

### Body composition and anthropometric measures

Body composition changed significantly over time. Baseline median body mass was 76.3 kg, BMI 25.83 kg/m2. At 6 months, body mass fell 15.2% (− 10.01 kg, *p* < 0.001) and BMI 12.5% (− 3.22, 95% CI − 4.96 to − 1.49, *p* < 0.001). Fat mass decreased 33.5% and fat % 22.4% (− 5.36, 95% CI − 7.92 to − 2.81, *p* < 0.001). Fat-free mass declined slightly (− 2.36, *p* = 0.058), total body water decreased (− 2.42, *p* = 0.007). At 12 months, body mass and BMI partially recovered (BMI − 0.46, *p* = 0.634; rebound: body mass p_adj_ = 0.0013, BMI padj = 0.014). Fat % exceeded baseline (+ 6.13, *p* < 0.001; recovery − 11.49, p_adj_ < 0.001). Fat-free mass remained stable (− 0.37, *p* = 0.765), total body water declined modestly (− 1.80, *p* = 0.043) without significant 6–12 month contrast (p_adj_ = 0.857).

### Predicted muscle mass, bone tissue mass, skeletal muscle mass, visceral fatty tissue, skeletal muscle index, metabolic age

At baseline, predicted muscle mass was 53.00 kg (IQR 47.50–57.99), skeletal muscle mass 31.60 kg (IQR 28.30–34.53), SMI 10.80 (IQR 10.03–12.08), bone mass 2.80 kg (IQR 2.52–3.02), visceral fat 10.00 (IQR 9.00–11.00), and metabolic age 53 years (IQR 52.00–54.04). Predicted muscle mass declined − 2.25 kg at 6 months (*p* = 0.058) and − 0.35 kg at 12 months (*p* = 0.764); skeletal muscle mass − 1.33 kg (*p* = 0.057) and − 0.92 kg (*p* = 0.187); bone mass and SMI showed no change. Visceral fat declined at 6 months (− 2.66, *p* < 0.001) with slight rebound at 12 months (+ 0.58, *p* = 0.194); age correlated positively (0.05, *p* = 0.004). Metabolic age decreased at 6 months (− 5.86, *p* < 0.001) and increased at 12 months (+ 2.98, *p* = 0.008); age correlation 0.19 (*p* < 0.001), no sex effect (1.34, *p* = 0.170). Contrast analysis confirmed recovery in visceral fat (− 3.23, p_adj_ < 0.001) and metabolic age (− 8.84, p_adj_ < 0.001), while SMI remained stable (− 0.41, p_adj_ = 0.910).

### Quality of life, sarcopenia and activity

At baseline, median QoL score was 62.11 (IQR 57.77–69.48), with 62.16% inactive. Weekly effort in METs was 441.48 (IQR 33.00–767.15). Moderate sarcopenia affected 32.43%. At 6 months, QoL improved to 73.07 (+ 17.6%), physical activity rose (13.51% active), METs increased to 1,640.83 (+ 2.72-fold), and sarcopenia affected 45.94%. At 12 months, QoL declined to 69.87 (− 4.4% vs 6 months, + 12.5% vs baseline), inactivity rose to 91.89%, METs fell to 1,021.86 (− 37.7% vs 6 months), and sarcopenia affected 45.95% (5.41% severe). QoL improved significantly at 6 months (10.92, 95% CI 6.84–15.00, *p* < 0.001) and 12 months (6.19, 95% CI 2.11–10.27, *p* = 0.003) vs baseline. Age correlated negatively with QoL (− 0.20, 95% CI − 0.34 to − 0.06, *p* = 0.007); gender showed no difference (− 2.48, 95% CI − 6.06 to 1.09, *p* = 0.172). METs increased at 6 months (1,182.13, 95% CI 911.59–1,452.68, *p* < 0.001) and 12 months (504.26, 95% CI 233.71–774.80, *p* < 0.001) vs baseline. Contrast analysis showed no QoL difference between 6 and 12 months (estimate 4.73, SE 2.06, p_adj_ = 0.064), but METs declined significantly (estimate 678, SE 136, p_adj_ < 0.001), indicating reduced activity after the 6-month peak though still above baseline.

### Hematological parameters

At baseline, median hemoglobin was 12.10 g/dL (IQR: 9.50–13.30), hematocrit 36.60% (IQR: 29.70–39.10), and platelet count 275.00 × 10⁹/L (IQR: 212.00–347.00). At 6 months, hemoglobin rose to 12.20 g/dL, hematocrit to 36.79%, and platelets to 278.06 × 10⁹/L. By 12 months, hemoglobin reached 12.76 g/dL, hematocrit 38.22%, and platelets 319.00 × 10⁹/L. At that time, 56.76% of patients were anemic: 54.05% mild, 2.70% moderate.

Contrast analysis showed no significant changes from 6 to 12 months in hemoglobin (− 0.52, p_adj_ = 0.387) or hematocrit (− 1.25, p_adj_ = 0.451), but platelet count increased significantly (− 28.38, SE = 11, p_adj_ = 0.029). Chemotherapy was not linked to hematopoietic toxicity, and no cytopenia cases occurred.

### Nutritional markers (albumin, prealbumin, Prognostic Nutritional Index, lymphocyte count)

At baseline, albumin was 4.05 g/dL (IQR: 3.84–4.33), with 91.89% of patients well-nourished and 8.11% mildly malnourished. Prealbumin was 0.21 g/L (IQR: 0.19–0.25), with 62.16% well-nourished and 37.84% mildly malnourished. Total protein was 64.10 g/L (IQR: 61.04–68.60), with 64.86% below normal. PNI was 48.60 (IQR: 45.75–53.15): 40.54% no malnutrition, 40.54% mild, 13.51% moderate, 5.41% severe.

At 6 months, albumin dropped to 3.97 g/dL (IQR: 3.77–4.36), with unchanged malnutrition status. Prealbumin stayed at 0.21 g/L (IQR: 0.18–0.23). Total protein rose to 65.50 g/L (IQR: 63.03–66.60), with 45.95% normal. PNI was 48.40 (IQR: 45.81–51.95): 43.24% no malnutrition, 37.84% mild, 13.51% moderate/severe.

At 12 months, albumin increased to 4.31 g/dL (IQR: 4.24–4.39), a 6.4% rise, with all patients well-nourished. Prealbumin declined to 0.20 g/L (IQR: 0.19–0.21): 70.27% no malnutrition, 24.32% mild, 5.41% moderate. Total protein rose to 68.64 g/L (IQR: 67.67–69.70), with 86.49% normal. PNI reached 52.42 (IQR: 51.91–54.42): 86.49% no malnutrition, 5.41% mild, 5.41% moderate/severe; 2.70% had severe malnutrition.

Contrast analysis showed no significant change in albumin at 6 months (− 0.03, 95% CI: − 0.16 to 0.09, *p* = 0.613), but a significant increase at 12 months (+ 0.27, 95% CI: 0.15 to 0.40, *p* < 0.001), with a significant contrast between 6 and 12 months (− 0.30, SE = 0.06, padj < 0.001). Prealbumin remained stable at both 6 months (− 0.01, *p* = 0.400) and 12 months (− 0.01, *p* = 0.136), with no significant contrast (padj < 0.001), although age was associated with lower levels (− 0.00, *p* = 0.042).

Total protein showed no significant change at 6 months (+ 1.21, *p* = 0.116), but increased significantly at 12 months (+ 4.52, *p* < 0.001), with a strong contrast between time points (− 3.32, SE = 0.76, padj < 0.001). PNI remained stable at 6 months (− 0.47, *p* = 0.640), but increased significantly at 12 months (+ 2.91, *p* = 0.004), with a significant contrast (− 3.37, SE = 0.99, padj = 0.002). Age and gender were not significantly associated with albumin, total protein, or PNI levels.

Lymphocyte counts gradually increased over 12 months, from a baseline median of 1.58 × 10⁹/L (IQR: 1.40–2.10) to 1.69 × 10⁹/L at six months and 1.89 × 10⁹/L at twelve months, indicating a steady upward trend during recovery.

### Lipid profile

At baseline, the median triglyceride (TG) level was 1.45 mmol/L (IQR: 1.14–1.74), total cholesterol (CHOL) was 4.21 mmol/L (IQR: 3.90–4.60), HDL was 1.27 mmol/L (IQR: 1.07–1.32), and LDL was 2.52 mmol/L (IQR: 2.10–2.63). Based on cholesterol levels, 91.89% of patients were not malnourished, while 8.11% were.

At 6 months post-surgery, TG decreased by 12.4% to 1.27 mmol/L (IQR: 0.88–1.66), CHOL slightly declined to 4.13 mmol/L (IQR: 3.47–5.00), and malnutrition dropped to 2.70%. HDL rose slightly to 1.30 mmol/L (IQR: 1.20–1.36), and LDL remained stable at 2.54 mmol/L (IQR: 2.20–3.04).

At 12 months, TG dropped further to 1.04 mmol/L (IQR: 0.85–1.39), CHOL decreased significantly to 3.74 mmol/L (IQR: 3.00–4.22), and malnutrition rose to 10.81%. HDL improved to 1.40 mmol/L (IQR: 1.34–1.46), and LDL decreased to 2.03 mmol/L (IQR: 1.59–2.47), a 19.4% reduction from baseline.

Contrast analysis showed significant TG reductions at 6 months (− 0.16, *p* = 0.048) and 12 months (− 0.33, *p* < 0.001), though the difference between these time points was not significant (padj = 0.100). CHOL showed no change at 6 months (− 0.01, *p* = 0.971), but a significant decrease at 12 months (− 0.47, *p* = 0.013), with a significant contrast (padj = 0.037). HDL increased significantly at 12 months (+ 0.15, p < 0.001), with a significant contrast from 6 months (− 0.10, padj = 0.014). LDL showed no change at 6 months (+ 0.16, *p* = 0.255), but decreased significantly at 12 months (− 0.39, *p* = 0.008), with a strong contrast (padj = 0.004).

No significant associations were found between age or gender and any lipid parameters.

### Iron, vitamin B12, vitamin D

At baseline, iron levels were 9.34 μmol/L (IQR: 5.76–14.25), slightly below the normal range of 10–30 μmol/L. Six months after surgery, iron dropped by 21.3% to 7.35 μmol/L (IQR: 5.03–10.20), indicating worsening deficiency. At twelve months, iron rose to 12.88 μmol/L (IQR: 12.12–13.50), a 75.3% increase from six months and 37.9% above baseline, restoring levels to the lower normal range. Statistically, iron decreased significantly at 6 months (− 2.37, *p* = 0.006) and increased significantly at 12 months (+ 2.76, *p* = 0.002), with significant differences from baseline (padj = 0.015 and padj = 0.003). Age and gender had no significant influence.

Vitamin B12 levels were elevated at baseline, measuring 1,136.09 pmol/L (IQR: 416.00–1,576.51), above the normal range of 450–1,000 pmol/L. At six months, B12 increased to 2,406.36 pmol/L (IQR: 736.36–3,645.60), a 1.12-fold rise. By twelve months, levels dropped sharply to 400.29 pmol/L (IQR: 365.00–431.89), an 83.4% decrease from six months and 64.8% below baseline, resulting in subnormal levels. Statistically, there was a significant increase at 6 months (+ 1227.33, *p* < 0.001) and a significant decrease at 12 months (− 634.99, p = 0.003), with a strong contrast between time points (estimate = 1862, padj < 0.001). No significant associations with age or gender were found.

Vitamin D levels were deficient at baseline at 21.84 ng/mL (IQR: 14.40–22.50), below the normal range of 30–50 ng/mL. At six months, levels rose slightly to 24.44 ng/mL (IQR: 22.46–26.22), a 12% increase but still suboptimal. At twelve months, levels increased to 33.04 ng/mL (IQR: 21.07–42.01), a 35.2% rise from six months and 51.3% above baseline, reaching the lower normal limit. Statistically, the change at 6 months was not significant (+ 3.37, *p* = 0.163), but the increase at 12 months was significant (+ 11.26, *p* < 0.001), with a significant contrast between time points (− 7.89, padj = 0.0031). Age and gender had no significant impact.

### Inflammatory and immune markers

At baseline, the neutrophil-to-lymphocyte ratio (NLR) was 2.83 (IQR: 2.06–3.62), with 72.97% of patients classified as having high NLR and 27.03% as low. The systemic immune-inflammation index (SII) was 778.99 cells/μL (IQR: 519.34–1,064.77), with 67.57% of patients in the high SII category and 32.43% in the low group.

Statistical analysis showed that NLR significantly decreased at 6 months (− 0.53, 95% CI: − 0.94 to − 0.12, *p* = 0.012) and at 12 months (− 0.45, 95% CI: − 0.86 to − 0.04, *p* = 0.033) compared to baseline. The contrast between 6 and 12 months was also significant (estimate = − 7.89, SE = 2.4, padj = 0.003), indicating a consistent downward trend. Age and gender had no significant impact on NLR (age: *p* = 0.188; gender: *p* = 0.250).

For SII, no significant changes were observed at 6 months (− 101.58, *p* = 0.083) or 12 months (+ 3.15, *p* = 0.957) compared to baseline. The contrast between 6 and 12 months was not significant (− 104.73, padj = 0.200). Age and gender also showed no significant associations with SII levels (age: *p* = 0.817; gender: *p* = 0.581).

## Discussion

The relatively small sample size (*n* = 37) inherently limits statistical power. Although several variables reached statistical significance, the large number of comparisons increases the risk of type I error. While the study was guided by the hypothesis that gastrectomy and perioperative treatment affect the metabolic and nutritional status of patients with gastric cancer, the limited existing evidence in this field and the broad range of evaluated parameters support an exploratory interpretation. Consequently, the findings should be interpreted with caution and the results are best viewed as hypothesis-generating and may inform the design of future, adequately powered confirmatory studies.

Treatment heterogeneity represents an additional limitation of this study. Patients were exposed to varying chemotherapy strategies, including preoperative, perioperative, postoperative, or no systemic treatment, with 19 patients receiving FLOT and only a subset continuing this regimen postoperatively, while others received unspecified protocols. This variability may have influenced metabolic and clinical trajectories and should be considered a potential confounding factor when interpreting the results*.* As assessments were conducted at the time of surgery, we are unable to directly evaluate the impact of preoperative chemotherapy on nutritional parameters; however, the postoperative data allow for indirect characterization of the patient group following neoadjuvant treatment.

Prehabilitation is increasingly recognized in perioperative care, with nutritional prehabilitation improving postoperative outcomes and preserving nutritional status in gastric, pancreatic, and colorectal cancers [18]. Preoperative chemotherapy provides a window for targeted nutritional interventions to enhance patient resilience. This non-metastatic cohort spanning stages IA–IIIB permits evaluation of clinical and nutritional parameters across disease severity, enabling assessment of tumor stage effects on treatment response, perioperative risk, and the efficacy of prehabilitation in a curative-intent setting.

### Body composition and anthropometric measures

Patients had a median body mass of 76.30 kg and BMI of 25.83 kg/m2, placing most in the overweight category per WHO criteria (BMI 25.0–29.9 kg/m2) [19]. Obesity is an established risk factor for gastric cancer, associated with chronic inflammation, insulin resistance, and hormonal dysregulation [20] — particularly in the context of GERD and metabolic dysfunction—and, being more prevalent than undernutrition, substantially influences nutritional status during oncologic treatment. At 6 months, body mass and BMI decreased significantly, driven by fat loss with minor lean mass reduction, consistent with postoperative catabolism [21]. Early weight loss is predominantly adipose, while muscle preservation depends on protein intake and rehabilitation [22]; high-protein diets may help reduce lean mass loss [23]. By 12 months, partial weight regain indicated an anabolic shift, but disproportionate fat gain relative to lean recovery suggests persistent nutritional and metabolic imbalance with potential long-term impact. Such weight regain in this population may reflect preferential restoration of adipose tissue rather than true anabolic recovery.

### Predicted muscle mass, bone tissue mass, skeletal muscle mass, visceral fatty tissue, skeletal muscle index, metabolic age

Baseline data showed prevalent central obesity, a risk factor for metabolic disease and gastric cancer, particularly with obesity-related inflammation and hormonal imbalance [24]. Postoperatively, skeletal and predicted muscle mass remained stable while visceral fat declined, contrasting with reports of muscle depletion after gastric cancer surgery [25]. Bone mass decreased, likely due to nutritional deficits and reduced mechanical loading [14].

Metabolic age improved from 53 to 47 years at 6 months, likely due to fat loss, though this may reflect malnutrition rather than true metabolic health [26]. The first 6 months showed expected catabolic effects, while by 12 months, muscle and bone partially recovered. However, visceral fat increased by 10%, and metabolic age rose above baseline, indicating unfavorable body composition changes. This fat rebound may stem from dietary shifts, reduced activity, or metabolic adaptation [27]

### Quality of life, sarcopenia and activity

Preoperative quality of life (QoL) in gastric cancer patients was moderate, probably impacted by disease burden, treatment side effects, and recovery challenges, as physical, emotional, and social well-being often decline due to fatigue, nutritional deficits, and psychological stress after diagnosis [27].

QoL improved at six months post-op, reflecting symptom relief and adaptation, but declined slightly at twelve months, likely due to dietary restrictions and fatigue. Physical activity rose at six months, though muscle strength lagged—likely due to nutritional limitations. At twelve months, activity dropped sharply, consistent with prior studies [28], and may reflect fatigue, sarcopenia, or insufficient rehab.

Sarcopenia—loss of skeletal muscle mass and strength—is associated with aging, chronic illness, and cancer [14], and, driven by metabolic changes, low protein intake, and impaired regeneration, contributes to functional decline despite mobility gains [29]. In the presented study, several methodological limitations concerning sarcopenia should be acknowledged. First, the assessment of skeletal muscle mass was based on bioimpedance analysis (TANITA SC-240), which, although practical and non-invasive, is less precise than computed tomography–based quantification at the L3 vertebral level and may be influenced by hydration status and other physiological variables. Second, the operational definition of sarcopenia in this study relied exclusively on muscle mass thresholds without incorporating functional measures such as handgrip strength or physical performance. In light of contemporary consensus definitions, which emphasize the integration of both quantitative and functional criteria, this approach may limit the accuracy and clinical relevance of sarcopenia classification [14]. The worsening of sarcopenia and reduced activity at twelve months underscore the need for structured exercise programs [28, 30].

Declining MET levels [31] and muscle health point to inadequate recovery and long-term surgical effects, reinforcing the importance of sustained rehabilitation and nutritional support.

MET levels and quality of life exhibited comparable trajectories, with the latter being less pronounced. This pattern may reflect an initial phase of structured postoperative rehabilitation, followed by attenuation of sustained behavioral engagement over the longer term. All patients were treated at a specialized referral center emphasizing perioperative nutrition and postoperative care, an approach that may enhance resilience to sarcopenia and treatment-related complications in upper gastrointestinal surgery. Recent evidence supports the efficacy of structured nutritional strategies, such as night home enteral nutrition (N-HEN), in mitigating postoperative weight loss and improving biochemical nutritional markers. In gastric cancer patients following total gastrectomy, N-HEN was associated with significantly reduced body weight loss, improved prealbumin levels, and enhanced tolerance to adjuvant chemotherapy, without increasing the risk of nocturnal glycemic disturbances. These findings underscore the importance of integrating targeted nutritional support into routine oncological care [32].

### Hematological parameters

Anemia in gastric cancer patients is mainly caused by inflammation, nutritional deficiencies, and impaired iron absorption due to gastrointestinal dysfunction [31]. After gastrectomy, iron deficiency anemia (IDA) and anemia of chronic disease (ACD) are common [33], and low preoperative hemoglobin is linked to worse outcomes, emphasizing early nutritional support [34]. Hemoglobin and hematocrit rose progressively over 12 months, indicating recovery, though mild anemia remained common, warranting continued monitoring and support.

### Nutritional markers

Albumin level is linked to inflammation and poor surgical outcomes when low [35], while prealbumin reflects short-term nutritional changes and correlates with long-term survival after gastrectomy [36]. However, serum albumin should be interpreted with caution as solely a nutritional marker, as its concentration reflects not only protein intake but also systemic inflammation and fluid balance. Albumin behaves as a negative acute-phase reactant and is substantially influenced by dilutional effects, limiting its specificity for nutritional status [37]. The Prognostic Nutritional Index (PNI), which combines albumin and lymphocyte count, is a predictor of postoperative complications and survival, with lower scores indicating worse outcomes [38].

In this study, albumin, total protein, prealbumin, and PNI levels showed consistent recovery over 12 months. Albumin and total protein rose markedly from 6 to 12 months, with most values normalizing. Prealbumin remained stable, indicating improved protein metabolism. PNI increased, reflecting reduced malnutrition, and the decline in moderate–severe cases suggests effective nutritional adaptation.

### Lipid profile

Dyslipidemia is frequent in gastric cancer [39], yet baseline lipid values here were normal. Over 12 months, lipid profiles shifted, and a slight rise in cholesterol-based malnutrition at 12 months highlights the need for ongoing nutritional monitoring. Adequate lipid intake and absorption remain important for long-term metabolic health. Low TC and LDL-C correlate with peritoneal metastasis, whereas higher LDL-C is linked to lung metastases in advanced disease [40].

### Iron, vitamin B12, vitamin D

Iron, vitamin B12, and vitamin D deficiencies are well-recognized consequences of gastrectomy, primarily due to impaired absorption and altered gastrointestinal physiology [21]. Iron deficiency anemia and vitamin B12 depletion are largely attributable to reduced gastric acid and intrinsic factor production, whereas vitamin D deficiency is associated with malabsorption and insufficient dietary intake [41]. In the present study, supplementation protocols were not standardized and varied in both dosage and route of administration, which should be considered when interpreting these findings.

At baseline, iron levels were slightly below the normal range, vitamin B12 levels were elevated—likely reflecting preoperative supplementation—and vitamin D levels were deficient. At six months, iron levels declined further, vitamin B12 increased, and vitamin D showed a modest, non-significant improvement. By twelve months, iron levels returned to the lower normal range; however, the persistence of anemia in a substantial proportion of patients suggests that restoration of circulating iron may not fully reflect functional iron status. The absence of detailed markers such as ferritin and transferrin saturation, as well as limited information on iron supplementation strategies, constrains the clinical interpretation of these findings.

Vitamin B12 levels decreased markedly at twelve months, reaching subnormal values, which is consistent with progressive depletion of intrinsic factor and underscores the need for long-term monitoring and supplementation. In contrast, vitamin D levels improved significantly despite not being routinely prescribed, which may reflect unrecorded self-supplementation, seasonal variation in sunlight exposure, or increased postoperative physical activity. Collectively, these observations highlight the importance of standardized supplementation protocols and comprehensive nutritional monitoring in this patient population [42].

### Inflammatory and immune markers

Elevated neutrophil-to-lymphocyte ratio (NLR) has been associated with poorer survival in gastric cancer and is considered a marker of systemic inflammation [43]. It may also have predictive value for immunotherapy response in advanced disease [44]. In the present study, 72.97% of patients exhibited elevated NLR preoperatively, which decreased to a median of 2.33 at six months, with prevalence declining to 59.46%, suggesting a reduction in inflammatory burden. A slight increase observed at twelve months may reflect persistent low-grade inflammation or adaptive immune processes; however, the clinical significance of these changes remains uncertain.

The systemic immune-inflammation index (SII), regarded as a more comprehensive prognostic marker than NLR or PLR, has been linked to advanced disease stages [45]. In this cohort, 67.57% of patients had elevated SII at baseline, with no significant changes over time. Although a postoperative decrease in NLR and an increase in PNI were observed, these findings should be interpreted with caution, as their clinical relevance cannot be fully established in the absence of correlations with disease stage, recurrence, or nutritional parameters. Obtaining such data would require long-term follow-up of the patient cohort.

Given the median age of 66 years in our cohort, the observed metabolic and inflammatory trajectories should be interpreted within the broader context of aging-related biological processes. Emerging evidence highlights the role of the aging–inflammation–cancer axis, characterized by chronic low-grade inflammation (“inflammaging”), immune remodeling, and impaired regenerative capacity, in shaping postoperative recovery. These mechanisms may partially explain the patterns observed in our study, including the initial reduction and subsequent partial rebound of NLR, the persistence of sarcopenia despite weight normalization, and the preferential regain of adipose tissue over lean mass. Such findings suggest that, beyond surgical and nutritional factors, age-associated alterations in inflammatory signaling and tissue homeostasis may contribute to the limited anabolic recovery and sustained metabolic imbalance in this population, warranting further investigation in future studies [46].

### Extending the scientific perspective

In upper gastrointestinal (UGI) surgery, nutritional assessment appears to be more consistently incorporated into clinical practice compared to other sub-specialties, with over 70% of surgeons evaluating parameters such as albumin levels and weight loss. However, despite this relatively higher awareness, the use of standardized screening tools remains limited and nutritional interventions are not systematically adapted to identified risk factors [47]. Recent bibliometric evidence highlights the growing scientific interest in immunonutrition and metabolic support in oncology, with increasing emphasis on their role in improving clinical outcomes and postoperative recovery. The present study aligns with this trend, contributing data from a Polish cohort and reinforcing the relevance of integrated nutritional assessment and intervention in patients undergoing gastrectomy for gastric cancer [48]. Despite the recognized importance of nutritional status in oncological outcomes, recent survey data indicate that a substantial proportion of clinicians managing gastrointestinal cancers do not adhere to standardized nutritional care protocols, with only about half being aware of validated approaches to diagnose and treat malnutrition. Moreover, nutritional assessment is inconsistently timed across the treatment trajectory. In this context, the present prospective study provides clinically relevant evidence by systematically evaluating metabolic and nutritional changes in a well-defined cohort of gastric cancer patients, thereby addressing an important gap and supporting the need for structured, longitudinal nutritional monitoring in routine practice [49].

## Conclusions

Most published studies on the metabolic effects of gastric cancer treatment have focused on patients undergoing surgery shortly after diagnosis, often with minimal preoperative preparation. The introduction of preoperative chemotherapy for the majority of patients with resectable gastric cancer has created a valuable time window for patient optimization, including nutritional support. However, chemotherapy itself may negatively affect appetite and nutritional status, underscoring the importance of studies addressing these dynamics. In our study, the prevalence of severe malnutrition among patients was low, with 5.41% affected at baseline and at the 6-month follow-up, decreasing to 2.70% at 12 months post-enrollment. One year after surgery, levels of albumin, total protein, HDL cholesterol, serum iron, and vitamin D showed significant increases compared to baseline values. In contrast, total lymphocyte count (TLC) and prealbumin levels remained statistically unchanged over the 12-month period. Notably, triglycerides, total cholesterol, LDL cholesterol, and vitamin B12 levels declined after 12 months. These findings suggest that nutritional supervision before and after gastric cancer resection may be critical for improving treatment outcomes.

## Supplementary Information

Supplementary Material 1.

Supplementary Material 2.

## Data Availability

No datasets were generated or analysed during the current study.

## Abbreviations

WHO: World Health Organisation
TLC: Total lymphocyte count
RR: Relative risk
CI: Confidence interval
ERAS: Enhanced Recovery After Surgery
BMI: Body mass index
QoL: Quality of life
METs: Metabolic Equivalent of Task
WBC: White blood count
PLT: Platelet count
PNI: Prognostic nutritional index
HDL: High-density lipoprotein
LDL: Low-density lipoprotein
NLR: Neutrophil-to-lymphocyte ratio
SII: The systemic immune-inflammation index
IQR: Interquartile range
