# Supplementary material for: Metabolic and nutritional impact of gastrectomy and perioperative treatment in gastric cancer: a prospective cohort study
Source: J Egypt Natl Canc Inst. 2026 Jun 22;38:36. doi: 10.1186/s43046-026-00367-6 (PMC13313303; doi:10.1186/s43046-026-00367-6)
Supplement: Supplementary file 1 — Supplementary Material 1. [file 43046_2026_367_MOESM1_ESM.docx]

**Supplementary material 1.**

**Detailed statistical methods description.**

The **significance level of α = 0.05** was established to allow for a 5% risk of Type I error. The distributions of numerical parameters were reported as median values (*Mdn*) along with interquartile ranges (*IQR: Q1, Q3*). For categorical variables, frequencies within each category (*n*) were reported alongside percentages alongside with the cohort size (*N*).

A robust linear mixed-effects model (robust LMM) was employed to analyze the changes within the studied clinical parameter over time with adjusting for confounder variables, while accounting for within-subject correlation through the inclusion of a random intercept for subjects. To reduce the influence of outliers in both residuals and random effects, the model was fitted using the robust fitting method DAStau. Optimization was performed using the "bobyqa" algorithm, with a maximum of 100,000 iterations to ensure convergence (Koller, 2016).

Robustness in the residuals and random effects was achieved using smoothed Huber-type rho functions, following established robust statistical procedures (Huber, 1981; Yohai, 1987). Residual robustness was implemented to mitigate the influence of extreme observations by applying robustness weights derived from smoothed Huber rho functions. These weights were calculated as a function of the residuals, where observations with larger residuals received smaller weights, reducing their influence on parameter estimation. This mechanism allowed the model to maintain efficiency while being resistant to the presence of outliers in the data (Maronna et al., 2006; Hampel et al., 1986). The approach ensured that the majority of observations contributed fully to the model, while extreme values were appropriately down-weighted.

Similarly, robustness weights were applied to the random effects variance component to minimize the influence of individual-level outliers. The random effects robustness weights were also generated using smoothed Huber rho functions, which adjusted the contributions of individual random effects based on their deviation from the group-level model. This mechanism down-weighted extreme random effects, limiting their impact on the variance component estimation and improving the model's robustness at the subject level (Koller, 2016; Schielzeth & Nakagawa, 2013).

Scaled residuals were computed to evaluate the fit of the model, ensuring that residuals were appropriately centered and standardized. Robustness weighting in the model allowed for the simultaneous handling of outliers in both the residuals and random effects, enhancing the reliability of parameter estimates in the presence of influential data points. The combination of residual and random effects robustness ensured a model fit that was resistant to deviations from normality and potential data contamination, as recommended in robust regression methodologies (Huber, 1981; Maronna et al., 2006). This robust modeling framework is increasingly recognized as a powerful approach for mixed-effects modeling of hierarchical data subject to outlier contamination (Groll & Tutz, 2014; Yohai & Zamar, 1997).

Changes between the reference level (pre-operative baseline) and the other time points (post-operative follow-up at 6 and 12 months) were evaluated using the results of the fitted regression model. Changes over post-operative time points were estimated through contrast analysis of the estimated marginal means, averaged over covariates to account for potential confounding effects. To control for multiple comparisons, p-values were adjusted using the Sidak correction method (Sidak, 1967). The p-values, adjusted p-values (*p_adj_*), and confidence intervals (*95% CI*) were derived based on an asymptotic approximation of the t-statistic.
