# Supplementary material for: Metabolic and nutritional impact of gastrectomy and perioperative treatment in gastric cancer: a prospective cohort study
Source: J Egypt Natl Canc Inst. 2026 Jun 22;38:36. doi: 10.1186/s43046-026-00367-6 (PMC13313303; doi:10.1186/s43046-026-00367-6)
Supplement: Supplementary file 2 — Supplementary Material 2. [file 43046_2026_367_MOESM2_ESM.docx]

**Supplementary material 2.**

**Abbreviations**

WHO – World Health Organisation

TLC - Total lymphocyte count

RR – relative risk

CI – confidence interval

ERAS - Enhanced Recovery After Surgery

BMI *-* Body mass index

QoL – quality of life

METs - Metabolic Equivalent of Task

WBC- white blood count

PLT- platelet count

PNI – prognostic nutritional index

HDL - high-density lipoprotein

LDL - low-density lipoprotein

NLR - neutrophil-to-lymphocyte ratio

SII - The systemic immune-inflammation index

IQR - interquartile range
